# Genome Sequences of Two Bacillus Phages Isolated from Indonesia

**DOI:** 10.1128/MRA.01058-19

**Published:** 2019-12-12

**Authors:** Yoga A. Handoko, Agustin K. Wardani, Aji Sutrisno, Simon B. Widjanarko, Trever L. Thurgood, Daniel W. Thompson, Ruchira Sharma, Julianne H. Grose

**Affiliations:** a Department of Agroindustry Technology, Brawijaya University, Malang, East Java, Indonesia; b Department of Agrotechnology, Satya Wacana Christian University, Salatiga, Central Java, Indonesia; c Department of Microbiology and Molecular Biology, Brigham Young University, Provo, Utah, USA; University of Southern California

## Abstract

Here, the genome sequences of two soil bacteriophages isolated from a red chili plantation in Indonesia are presented. The genome of vB_BspS_SplendidRed (42,859 bp) is highly similar to Bacillus phage Ray17 from the United States, while vB_BspM_MarvelLand (156,945 bp) is highly similar to *Bacillus* phage BC01 from South Korea.

## ANNOUNCEMENT

Bacillus spp. are rod-shaped, Gram-positive, aerobic bacteria capable of forming endospores when exposed to extreme environments (1–3). In the agroindustry, some species of *Bacillus*, such as B. subtilis, B. thuringiensis, B. licheniformis, B. pumilus, B. anthracis, and B. cereus, can contaminate raw material and food products (4, 5). *Bacillus* spp. can encode saprophytic enzymes such as α-amylases and proteases, which can damage bread products, pasta, cocoa, milk, meat products, and chili-based products, including chili bo (6–12). Bacteriophages (phages) are viruses that can infect and kill bacteria (4, 13), making them prime candidates for treating bacterial diseases, including *Bacillus*-related disease (14).

Herein, we announce the genome sequences of two novel *Bacillus* phages, vB_BspS_SplendidRed (SplendidRed) and vB_BspM_MarvelLand (MarvelLand). These phages were isolated from soil collected from a red chili plantation in Getasan Village, Semarang District, Central Java State, Indonesia (global positioning system coordinates 7°23′09.9″S, 110°27′35.1″E). The *Bacillus* sp. environmental host was isolated from decayed red chili fruit (Capsicum annuum L.) by plating onto lysis broth (LB) plates, and it was identified as a *Bacillus* sp. by using 16S rRNA sequencing (with primers described by Liu et al. [15]). The phages were isolated by propagation in an enrichment culture, followed by plaque purification from top agar (adapted from Czajkowski et al. [16] and Sauder et al. [17]).

Phage genomic DNA was extracted using the Norgen Biotek phage DNA isolation kit. The Illumina TruSeq Nano DNA kit was used for genomic library preparation, with each phage having unique barcodes, followed by sequencing on the Illumina HiSeq 2500 platform (250-bp paired-end reads). Geneious version 8.0.5 (18) reported one circular contig for each phage from the *de novo* assembly algorithm, using 20% of the 1,275,056 reads for MarvelLand (with no preassembly quality checking) and 90% of the 1,220,418 reads for SplendidRed (with the ends trimmed preassembly); the percentage of reads used was determined empirically to achieve a circular contig. The Geneious-based assembly of MarvelLand resulted in two ambiguities (base pairs 31070 and 31095) that had differential base calling. These ambiguities were resolved by PCR amplification of the region (base pairs 30985 to 31355), followed by Sanger sequencing, which confirmed the most prominent base pairs identified by Illumina sequencing. Phages were annotated using DNA Master (19), giving preference for calls that gave full coding potential coverage (20, 21). Default parameters were used for all software unless otherwise stated.

Analysis of the phage genome sequences suggests that SplendidRed (42,859 bp) is a new member of the *Caudovirales Siphoviridae* family, having 93% nucleotide similarity by BLASTN (22) to *Bacillus* phage Ray17, isolated in the United States (23). MarvelLand (156,945 bp) is likely a member of the *Caudovirales Myoviridae* family, having 94% nucleotide similarity by BLASTN to *Bacillus* phage BC01, isolated in South Korea (24). The SplendidRed and MarvelLand genome termini were determined by BLASTN alignment to Ray17 and BC01, respectively. The current annotation suggests that SplendidRed encodes at least 76 open reading frames (ORFs), while MarvelLand encodes 241 ORFs and 18 tRNAs. These phages encode proteins with predicted functions in DNA metabolism, virion structure, packaging, and cell lysis.

### Data availability.

The GenBank and SRA accession numbers for the *Bacillus* phages SplendidRed and MarvelLand are listed in Table 1.

**TABLE 1:** Basic properties of the two *Bacillus* phage genomes

Phage name	GenBank accession no.	SRA accession no.	Min–max^*a*^ fold coverage (avg read depth)	Genome length (bp)	No. of ORFs	No. of tRNAs	G+C content (%)
vB_BspS_SplendidRed	MN013088	SAMN12262426	338–736 (517.2)	42,859	76	NA^*b*^	44.58
vB_BspM_MarvelLand	MN013089	SAMN12262427	1–1,882 (398.9)	156,945	241	18	40.19

a Min–max, minimum to maximum.

b NA, no tRNAs were identified.
